# Effects of subconscious and conscious emotions on human cue–reward association learning

**DOI:** 10.1038/srep08478

**Published:** 2015-02-16

**Authors:** Noriya Watanabe, Masahiko Haruno

**Affiliations:** 1 Center for Information and Neural Networks, National Institute of Information and Communications Technology, Suita, Osaka 565-0871, Japan; 2 Japan Society for Promotion of Science; 3 Graduate School of Environmental Studies, Nagoya University; 4 Japan Science and Technology Agency

## Abstract

Life demands that we adapt our behaviour continuously in situations in which much of our incoming information is emotional and unrelated to our immediate behavioural goals. Such information is often processed without our consciousness. This poses an intriguing question of whether subconscious exposure to irrelevant emotional information (e.g. the surrounding social atmosphere) affects the way we learn. Here, we addressed this issue by examining whether the learning of cue-reward associations changes when an emotional facial expression is shown subconsciously or consciously prior to the presentation of a reward-predicting cue. We found that both subconscious (0.027 s and 0.033 s) and conscious (0.047 s) emotional signals increased the rate of learning, and this increase was smallest at the border of conscious duration (0.040 s). These data suggest not only that the subconscious and conscious processing of emotional signals enhances value-updating in cue–reward association learning, but also that the computational processes underlying the subconscious enhancement is at least partially dissociable from its conscious counterpart.

To achieve our behavioural goals, we must continuously adapt our behaviour and learn from changing circumstances. However, the great majority of incoming signals in real-life social situations is irrelevant to our immediate goals, and may be processed unconsciously in many situations. An intriguing question is whether such irrelevant and subconsciously received information can affect behavioural adaptation.

Many studies report that emotional information not necessary for achieving an immediate task goal can affect aspects of human behaviour including decision making^1^, clarity of memory^2^, and learning rates during cue-reward association learning^3^, and that this is true even when the people are aware that the information is irrelevant to achieving the task goal. For instance, in a cue-reward association-learning study, presentation of a task-independent fearful face just before the reward-predicting cue accelerated the learning rates compared with presentation of a neutral face; an enhancement effect that was not found in a similarly designed short-term memory task^3^. However, all of these experiments employed an emotional signal that subjects could consciously perceive, and did not account for incoming information that is processed subconsciously (e.g. the surrounding social atmosphere such as feelings of tension in a classroom). Although shorter duration of stimulus presentation generally induces smaller behavioural effects and neuronal responses, some studies report that subconscious presentation of information or subconscious thought results in larger effects than does conscious counterpart^4^,^5^,^6^,^7^, and can affect human behaviour in daily life^8^,^9^. Therefore, it is important to clarify whether and how subconscious emotional information influences human learning.

Here, we performed a computational model-based analysis of behaviour to examine how learning of a probabilistic cue-reward association is affected when emotional facial expressions are shown subconsciously or consciously before presentation of the reward-predicting cue. We have previously found that learning was enhanced when the duration of face presentation was long (1.0 s)^3^ and thus focus here on how learning is affected by a duration (0.027–0.047 s) that yields less recognisable faces.

## Results

### Facial Discrimination task

**Figure 1:**
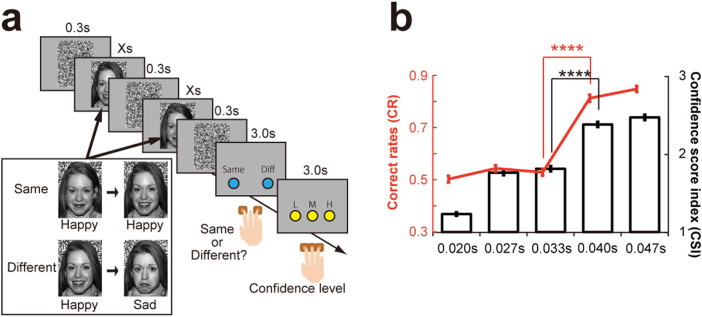
Task design and behavioural results for the discrimination task. (A) Two facial expressions (happy or sad) were presented sequentially with masks. Duration of each presentation was 0.020 s, 0.027 s, 0.033 s, 0.040 s, or 0.047 s. Participants were required to determine whether the presented expressions were the “same” or “different”, and to rate their confidence level (“low”, “medium”, or “high”). (B) Both the correct rate (CR: red) and confidence score index (CSI: black) (mean ± SEM) showed that the ability to discriminate facial expression sharply increased at 0.040 s (CR, paired t-test, t_(90)_ = −17.808, *p* < 0.001; CSI, paired t-test, t_(90)_ = −17.033, *p* < 0.001 with BC). *p < 0.05, **p < 0.01, ***p < 0.005, and ****p < 0.001 throughout the figures. This image is not covered by the [CC licence]. Photographs are from the NimStim Face Stimulus Set. Development of the MacBrain Face Stimulus Set was overseen by Nim Tottenham and supported by the John D. and Catherine T. MacArthur Foundation Research Network on Early Experience and Brain Development. (http://www.macbrain.org/resources.htm).

Before the main learning task, we conducted a discrimination task (n = 91) to estimate duration thresholds for conscious discrimination of facial expressions that were based on objective (correct rate) and subjective (confidence scoring) measures (Figure 1a). We regarded a presentation as ‘conscious' if it was delivered above both subjective and objective thresholds, and as ‘subliminal' if it was lower than both thresholds. We define ‘subconscious' presentation as being at a duration between subliminal and conscious presentations.

We conducted a series of t-tests to determine the threshold duration. Analysis showed that performance accuracy (the correct rate, [CR]) at a duration of 0.040 s was higher than at 0.033 s (paired t-test, t_(90)_ = −17.808, *p* < 0.001 with Bonferroni corrections [BC]), but not for any other comparisons (0.020 s vs. 0.027 s: t_(90)_ = −2.294, *p* = 0.360; 0.027 s vs. 0.033 s: t_(90)_ = 0.982, *p* ≈ 1.000; 0.040 s vs. 0.047 s: t_(90)_ = −2.470, *p* = 0.225 with BC) (Figure 1b, red; comparison among five durations). Additionally, although the CRs in 0.020 and 0.033 s were not different from chance level (paired t-test, 0.020 s: t_(90)_ = 0.156, *p* ≈ 1.000; 0.033 s: t_(90)_ = 2.250, *p* = 0.405 with BC), CR in 0.027 s was slightly and significantly higher than the chance level (paired t-test, t_(90)_ = 3.551, *p* = 0.015 with BC) (Figure 1b red).

Consistent with the CR analysis, the subjective confidence score index (CSI) showed that participants discriminated facial expressions when they were presented for longer than 0.040 s significantly better than at shorter durations (0.033 s vs. 0.040 s: paired t-test, t_(90)_ = −17.033, *p* < 0.0001 with BC) (Figure 1b black). While CSI comparisons did not differ significantly between 0.027 s and 0.033 s (t_(90)_ = −2.347, *p* = 0.211 with BC) or between 0.040 s and 0.047 s (t_(90)_ = −2.373, *p* = 0.199 with BC), they did differ significantly between 0.020 s and 0.027 s (t_(90)_ = −19.632, p < 0.001 with BC).

We also sorted CSIs based on task performance to confirm that participants rated their correct trials as more certain. We found that although CSIs at 0.020 s and 0.027 s stimulus durations did not differ between correct and error trials (paired t-test, 0.020 s: t_(90)_ = 1.577, *p* ≈ 1.000; 0.027 s: t_(90)_ = 1.549, *p* ≈ 1.000 with BC), they did differ at longer durations (paired t-test, 0.033 s: t_(90)_ = 6.012, *p* < 0.0001; 0.040 s: t_(90)_ = 8.981, *p* < 0.0001, 0.047 s: t_(90)_ = 8.564, *p* < 0.0001 with BC) (Supplementary Fig. S1).

These results showed that participants correctly discriminated facial expressions with high confidence at presentation durations of 0.040 s and 0.047 s, which thus represents conscious presentations as we defined them. In contrast, it was impossible to discriminate facial expressions either objectively or subjectively when faces were presented for only 0.020 s. The other two durations (0.027 s and 0.033 s) represent subconscious presentation because participants showed similar confidence levels in correct and error trials with better-than-random CR at 0.027-s durations, while at 0.033-s durations they could not discriminate faces objectively even with the high CSI in the correct trials. Based on these observations, we used 0.027 s or 0.033 s for the subconscious condition and 0.040 s or 0.047 s for the conscious condition in the learning task. This definition of the subconscious and conscious conditions is similar to that in other studies using facial expressions^10^,^11^.

In the learning task, each participant was randomly assigned to one of these four durations. To rule out other possible factors affecting learning performance, we assessed several individual differences including age, sex, the time we conducted the experiment, and intelligence level. We did not find any factor that was biased among the groups (Table 1, see *statistical analyses for sampling bias* section).

### Learning task

**Figure 2:**
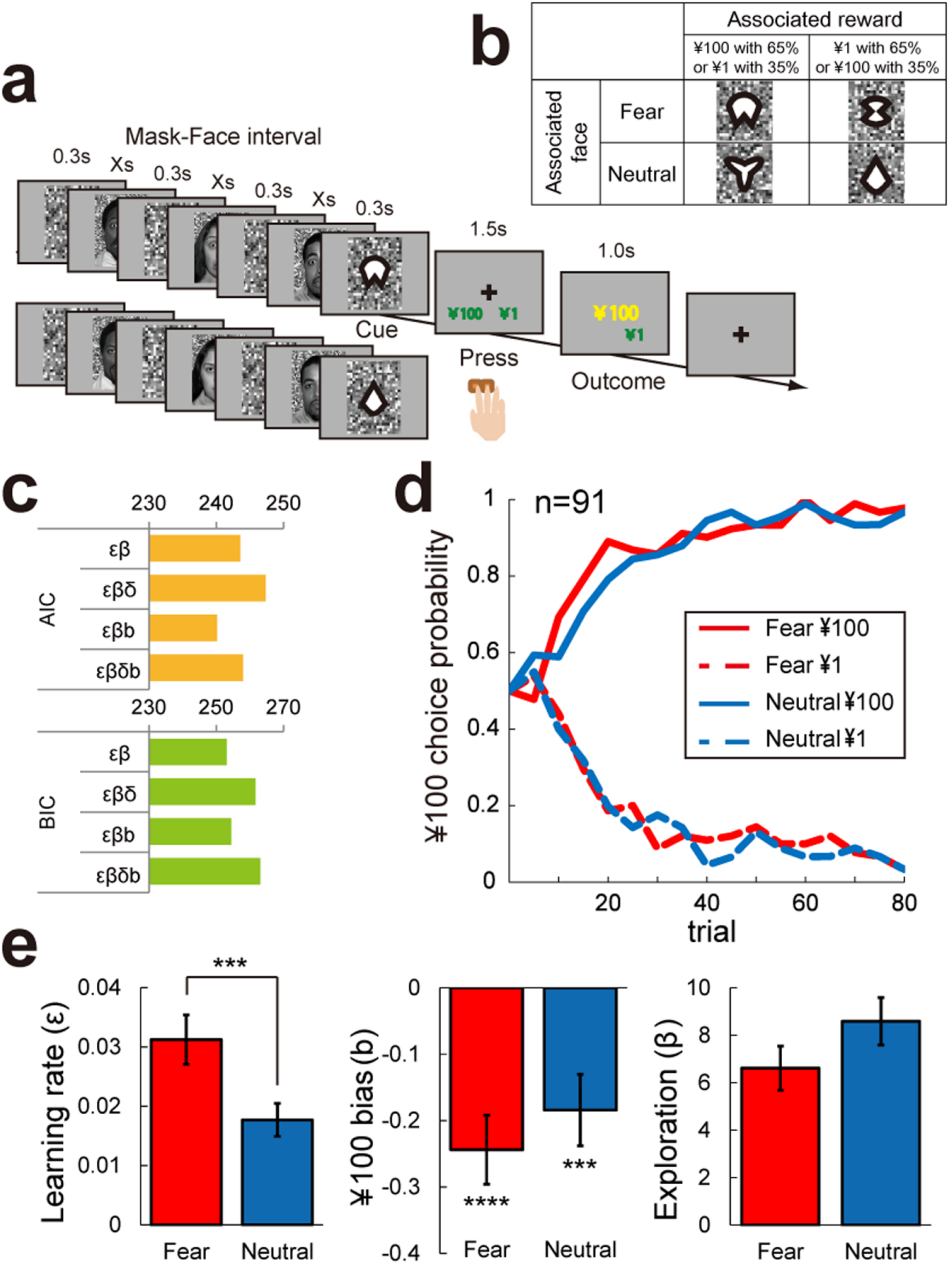
Task design, behavioural results, and model-based analysis of the learning task. (A) Participants were required to press a button to indicate which reward would they expected, and eventually learned the association between particular rewards and particular cues. The duration of face presentations was randomly assigned as 0.027 s, 0.033 s, 0.040 s, or 0.047 s for each participant. (B) An example combination of the facial expression, cue, and reward. Each of the four cues was associated probabilistically (65%) with one of the two different reward amounts, and also with one of the two facial expressions (fearful or neutral faces with 100% probability). (C) The results of the parameter estimation by AIC and BIC. ε, β, δ, *b* represent the learning rate, exploration, reward sensitivity, and ¥100 bias, respectively. (D) Learning curves. Each data point represents the average of five trials. (E) εβb model-based estimation of the learning rate, the ¥100 bias, and exploration (mean ± SEM). Photographs are from the NimStim Face Stimulus Set. Development of the MacBrain Face Stimulus Set was overseen by Nim Tottenham and supported by the John D. and Catherine T. MacArthur Foundation Research Network on Early Experience and Brain Development. (http://www.macbrain.org/resources.htm).

To examine the computational processes behind the interaction between reward learning and subconscious/conscious emotional processing (Figure 2a and 2b), we analysed behaviour using a reinforcement learning model. More specifically, we estimated the following four parameters. The learning rate (ε) controls reward prediction error in each trial. The exploration parameter (β) controls how deterministically a value function leads to advantageous behaviour, and reward sensitivity (δ) transforms the actual reward into a subjective reward, as the emotional stimulus can change subjective sensitivity to reward. The last parameter is a ¥100 choice bias (*b*), which is a value-independent bias for choosing the ¥100 option. This parameter represents the possibility that participants were biased to choose one of the two rewards depending on facial emotional expression, regardless of cue-reward associations. We estimated these parameters separately for fearful or neutral conditions (see *Reinforcement learning model-based analysis*). Before the detailed analysis, we quantified the appropriateness of our statistical models using Akaike information criteria (AIC) and Bayesian information criteria (BIC). As shown in Figure 2c, the *εβb* model, which includes learning rate, exploration parameter and ¥100 bias, was selected by AIC, and the *εβ* and *εβb* models were comparable using BIC (*εβ* was slightly better). Model comparisons were highly consistent with our previous report^3^ and we used the *εβb* model in subsequent analyses.

Learning curves averaged separately for each cue, irrespective of face presentation duration (n = 91), are shown in Figure 2d. Especially in the early stages, learning was faster for cues associated with fearful faces and the ¥100 reward than other cues (solid red line). To conduct a more quantitative analysis, we examined the effects of emotion (fear vs. neutral) on each parameter of the computational model (learning rates, ¥100 choice bias, and exploration). Consistent with our previous report with 1.0-s face presentations^3^, we found that the learning rate was higher in the fearful condition than in the neutral condition (t_(90)_ = 3.077, *p* = 0.003) (Figure 2e, left). Additionally, ¥100 choice bias was negative in the fearful condition (t_(90)_ = −4.687, *p* < 0.001 with BC), and no difference was found in the exploration parameter (t_(90)_ = −1.552, *p* = 0.124) (Figure 2e, middle and right). The only notable difference from our previous study was that ¥100 choice bias was negative in the neutral condition (t_(90)_ = −3.401, *p* = 0.002 with BC) (Figure 2e, middle).

**Figure 3:**
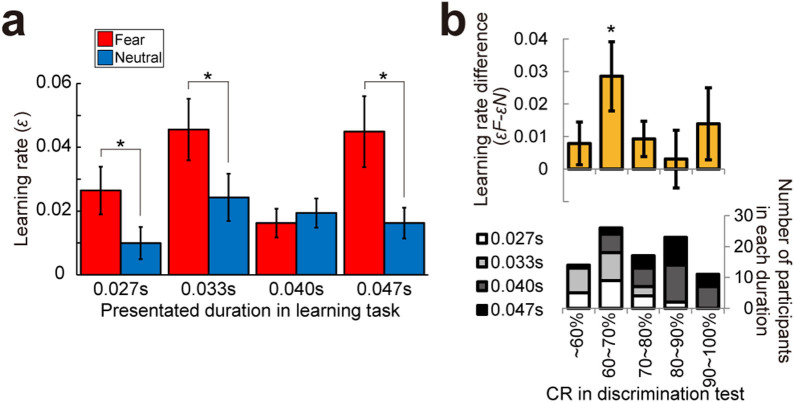
Average learning rates sorted by presentation duration and correct rate. (A) Learning rates sorted by presentation durations revealed a behavioural trough in the fearful condition at 0.040 s duration. Learning rate differences (εF−εN) for each duration were higher than zero (ts ≥ 2.194, *p*s < 0.05), except for the 0.040 s duration (t_(30)_ = −0.560, *p* = 0.580). (B) Learning rates sorted by correct rates (CRs) in the discrimination test (upper panel) showed that participants with 60%–70% CRs were most affected in terms of their learning rates (t_(25)_ = 2.628, *p* = 0.014). The lower panel refers to the number of the participants in each CR condition.

Having seen that emotional face presentation modulates the learning rates and ¥100 choice bias, we then investigated how subconscious presentation of emotional faces affects learning rates and the ¥100 choice bias. To achieve this, we separately computed learning rates for each presentation duration (0.027 s: n = 20; 0.033 s: n = 20; 0.040 s: n = 31; 0.047 s: n = 20) (Figure 3a). A two-way ANOVA (2 Emotions × 4 Presentations) showed a significant main effect of emotion (F_(1,87)_ = 13.306, *p* < 0.001) and no main effect of presentation duration (F_(3,87)_ = 2.508, *p* = 0.064). Importantly, the interaction between emotion and presentation duration was significant (F_(3,87)_ = 2.946, *p* = 0.037), suggesting that the learning enhancement provided by the fearful faces may disappear at some durations. Therefore, we looked into the effects of emotion on the learning rate of each presentation duration.

The learning rate differences (εF −εN) were larger than zero in the 0.027 s (t_(19)_ = 2.211, *p* = 0.040), 0.033 s (t_(19)_ = 2.482, *p* = 0.023), and 0.047 s (t_(19)_ = 2.194, *p* = 0.041) conditions, but not in the 0.040 s condition (t_(30)_ = −0.560, *p* = 0.580). This targeted analysis revealed a trough in the emotional enhancement effect at around 40 ms. To interpret this result in terms of perception, we sorted the subjects by CR scores on the discrimination task (mean ± SE CRs, ~60%: 0.478 ± 0.023; 60%–70%: 0.667 ± 0.007; 70%–80%: 0.757 ± 0.004; 80%–90%: 0.851 ± 0.006; 90%–100%: 0.960 ± 0.009) and found that the emotional face-induced increases in learning rates was strongest (n = 26, εF−εN = 0.028 ± 0.011) when the participants' CRs were 60%–70% (t_(25)_ = 2.628, *p* = 0.014) (Figure 3b), and the enhancement effect disappeared at around CRs of 70%–80% (n = 17, εF−εN = 0.009 ± 0.005, t_(16)_ = 1.655, *p* = 0.117) and 80%–90% CRs (n = 23, εF−εN = 0.003 ± 0.009, t_(22)_ = 0.341, *p* = 0.736). The increase in the learning rate caused by emotional faces started to be discernible again at CRs of 90%–100%, although this was not statistically significant (n = 11, εF−εN = 0.014 ± 0.011, t_(10)_ = 1.203, *p* = 0.257).

We conducted the same analysis for the ¥100 choice bias. A two-way ANOVA (2 Emotions × 4 Presentations) revealed a significant main effect of presentation duration (F_(3,87)_ = 3.287, *p* = 0.024), but not of emotion (F_(1,87)_ = 0.973, *p* = 0.327), or the interaction (F_(3,87)_ = 0.356, *p* = 0.785). Importantly, these data demonstrate that the trough was observed for the learning rates but not for the ¥100 bias.

## Discussion

In this paper, we used a computational model-based behavioural analysis of probabilistic cue–reward association learning to determine whether subconscious and task-independent emotional signals affect learning. We found that the learning rate for cues paired with a fearful face was larger than for cues paired with neutral faces, and that this enhancement effect was significant when the face was presented subconsciously (durations of 0.027 s or 0.033 s) and consciously (0.047 s). However, this effect disappeared at 0.040 s. Furthermore, not only does the effect of emotional signals on learning rates vanish at the presentation duration of 0.040 s, but this duration also corresponds to the 70%–90% CR level, validating the discontinuity of the learning-enhancement effect. Because we did not observe this effect in the discrimination task or in the ¥100 choice bias, it is likely to be specific to the associative learning paradigm.

The discontinuity of the learning-rate enhancement effect might have been caused by some malfunction in our experimental devices for stimulus presentation. However, if this was the case, we would expect the same problem to have occurred for the objective CRs in the discrimination task. As we did not observe any significant performance trough in Figure 1b at 0.040 s, and because almost all participants reported that they were conscious that two facial expressions were presented in the 0.040 s condition (post-experimental questionnaire), we can rule out the possibility of an experimental device-dependent problem. Another possibility is that the trough resulted from some sampling bias among different groups. However, we examined sex, age, experiment time, and academic scores (as shown in Table 1) and did not find any difference among the groups (see *Statistical analyses for sampling bias*).

One plausible explanation for the disappearance of the enhancement effect is that there are two pathways for emotional signal processing in the brain^12^,^13^. One system is the cortical pathway, which is routed through several visual stages such as the retina, lateral geniculate nucleus of the thalamus, primary visual area cortex, higher-order brain areas, and finally extending to the amygdala. This route of information processing results in precise perception in which we are conscious of presented stimuli. The other system is the subcortical pathway, which is routed through the retina, superior colliculus, pulvinar nucleus of the thalamus, and extends to the amygdala. Although information processing via this route is comparatively crude, it is thought to be an implicit system that works faster than the cortical pathway. Several behavioural and brain-imaging studies have shown that the subcortical pathway has sensitivity to the rapid presentation (faster than 0.033 s) of emotional facial expressions^11^,^14^,^15^. Therefore, the subconscious presentation (0.027 s and 0.033 s) of stimuli presented here could have driven the subcortical pathway, whereas the 0.047 s presentation drove the cortical pathway. These two systems could have different effects on reward-based learning systems that include the substantia nigra, ventral striatum, and amygdala as implicated in previous studies^3^,^11^,^14^,^15^,^16^,^17^.

Similar discontinuity effects observed in behavioural responses to visual stimuli have also been reported as the ‘performance-dip effect^5^,^6^, which is defined as the lowered accuracy in a main task when it is paired with the presentation of a para-threshold task-irrelevant stimulus. These experiments and our current observations are compatible in the sense that performance of the main task was affected when either a subconscious or clear task-irrelevant visual stimulus was presented. Importantly however, while previous experiments showed that the task-irrelevant stimuli reduced performance, our results showed the opposite effect: subconscious emotional signal enhanced learning.

One might wonder which enhances learning more, conscious or subconscious perception of emotional stimulus. Although the effects of subconscious stimulation tend to be weaker in general than conscious stimulation, some studies have reported that subconscious presentation of stimuli was more effective^4^,^5^,^6^. Here, we showed that enhancement by emotion perception was significant in both subconscious and conscious conditions, except when the stimulus duration was 0.040 s. However, as shown in Figure 3b, participants were most affected by the emotional signal when their accuracy was between 60% and 70%. This result seems to suggest that the learning-enhancement effect is strongest when the emotional signal is presented obscurely. Figure 3b also indicates that overly quick stimulus presentation (~60% CRs: mean CR = 0.478 ± 0.023) does not enhance learning rates. These results may indicate that there is an optimal range of presentation durations for emotional signals that yield subconscious enhancement of learning.

Finally, while the ¥100 choice bias (which was independent of learning) was also affected by presentation duration, no trough in the effect was observed. Although faces were unrelated to our main learning task, the subconsciously presented faces may have induced uncertainty^18^ or anxiety concerning subjective perception, and the negative feeling may have led to negative choices (smaller reward). Such a transfer of the task-independent feeling to the main task could well be linked with Pavlovian Instrumental Transfer (PIT)^19^,^20^. The PIT is a phenomenon in which previously conditioned Pavlovian cues affect the subjective prediction and motivation in subsequent instrumental conditioning from the outset, despite no explicit association between the Pavlovian cue and the new learning^19^,^20^. In the current learning experiment, the subconscious presentation of facial expressions could have induced negative emotion, and this emotion then transferred the subsequent associative learning from the very first trial. Such a negative bias might have been quantified as the negative ¥100 choice bias.

## Methods

### Participants

Participants in this study were undergraduate and graduate students who did not declare any history of psychiatric or neurological disorders. All experiments were conducted according to the principles in the Declaration of Helsinki and were approved by the ethics committee of the National Institute of Information and Communications Technology. All 130 participants gave informed consent prior to the experiments. Thirty-nine people (30.0%) were unable to learn all four of the associations. Therefore, we analysed data from the remaining 91 participants (64 male; mean age 21.5 ± 1.7 years).

### Experimental design

Stimuli were presented via a Dell precision T7500 computer with a graphics accelerator (NVIDIA Quadro 4000) and 19 inch CRT display (SONY CPD-G420) to achieve 150 Hz refresh rates. Stimulus presentation and response acquisition were controlled using Psychtoolbox-3 software (www.psychtoolbox.org) with MATLAB. Stimuli were presented within an area subtending 4.49 × 6.16 degrees of visual angle.

### Facial discrimination task

Prior to the learning task, all 91 participants performed the facial expression discrimination task (Figure 1a), which measured the presentation-time threshold for subconscious and conscious facial expression discrimination. We used 8 happy and 8 sad faces of the same 4 actors and 4 actresses including 6 Caucasoid, 1 Negroid, and 1 Mongoloid from the NimStim^21^ collection that have high validity and reliability of expressions. Three masks (presented for 0.3 s each) and two emotional faces (displayed for 0.020 s, 0.027 s, 0.033 s, 0.040 s or 0.047 s) were presented alternately on a screen (see Figure 1a). To maximise the effects in the main learning task, we did not use fearful or neutral faces in this task. We reasoned that prior knowledge of the facial expressions might affect participant's behaviour in the main learning task. Additionally, repetitive presentation of the same emotional pictures could lead to reduced stimulus saliency.

Participants were required to discriminate the two expressions of an identical actor or actress by answering whether the first expression was the “same” as the second one within 3.0 s. Participants indicated their answers by pressing a button with the right index (same) or ring (different) fingers. Additionally, they were asked to indicate how confident they were in their answers (“low confidence”, “medium confidence”, or “high confidence”) with the right index, middle, and ring fingers, respectively. As we used two different pictures of an identical actor or actress with forward and backward masks for each trial, participants could not judge the difference of expressions based on outlines of faces or afterimages. This task included 80 trials (8 same and 8 different trials ×5 presentation conditions in a pseudo-random order). As the participants were trained for several practice trials with another stimulus set (happy and sad faces), they executed this task flawlessly.

### Learning task

For the main learning task, participants learned probabilistic associations (65% or 35%) between four visual cues and two rewards (¥100 or ¥1) through trial and error (Figure 2a). The design was similar to a previous experimental paradigm^3^ except for the brief presentation of facial expressions. Each participant was randomly assigned to one of four face-presentation durations (0.027 s, 0.033 s, 0.040 s, or 0.047 s). We used a between-participants design for the four durations because of task difficulty and to avoid the effects of repetition, such as habituation to the task or meta-learning of task structure^22^.

Face stimuli were 20 fearful and 20 neutral faces of 10 actors and 10 actresses, including 10 Caucasoid, 7 Negroid, and 3 Mongoloid. Just before the visual cue (0.3 s), either a fearful or neutral face interleaved with four masks (0.3 s) was presented three times on a screen for an individually and randomly assigned duration in a pseudo-random order. Only one emotion was used within a given trial. Following the last face, one of the cues was presented, followed by a choice between ¥100 and ¥1. Participants then pressed a button within 1.5 s to indicate which of the two rewards they expected. The order of cue presentation and the assignment of the two buttons (left or right) with rewards were randomised across trials. After making their choice, the actual reward was shown in yellow letters for 1.0 s. Over time, participants could then learn the association between each cue and the corresponding reward. Before the experiments, we confirmed that the participants fully understood this task. They were instructed that the face and noise presentations would signal the appearance of a cue. No participants reported noticing any associations between particular facial expressions and the cues. The combinations of the four visual cues, facial expressions, and rewards were counterbalanced across participants (Figure 2b). The total number of trials was 320 (80 × 4 conditions).

### Statistical analyses for perceptive discrimination task

The correct rates (CR) were calculated by dividing the sum of the hit rate and correct rejection rate by the number of trials (16) (Figure 1b red). We calculated the subjective confidence level for each judgment using the confidence score index (CSI). For this index, each raw rating was 1, 2, or 3, representing “low confidence”, “medium confidence”, or “high confidence”, respectively. This rating was independent of the correctness of the judgment, and was averaged for each duration (1 ≤ CSI ≤ 3) (Figure 1b black). Additionally, we sorted the CSI data based on the CR (Supplementary Fig. S1).

### Statistical analyses for sampling bias

The learning task was conducted using a between-participants design for the four presentation durations to avoid fatigue, habituation, and meta-learning of task structure^22^. However, this might have induced sampling bias. We therefore examined four possible biases: age, sex, the time of day the experiment started, and the intelligence level based on university-department academic scores. The mean experimental start time was taken into account because experiments conducted in the early morning or late at night may be associated with different arousal levels, even though we reminded participants by email before participation to get enough sleep. Results are summarised in Table 1 and there was no bias in any of the four groups.

### Reinforcement learning model-based analysis

To conduct a trial-based analysis of the learning process, we adopted a reinforcement learning model^3^,^23^,^24^. This model assumes that each participant assigns the value function *Q_t_*(*s_t_*, *a_t_*) to action *a_t_* for the cue *s_t_* at time *t*. Learning increases the accuracy of value representation by updating the value in proportion to the reward prediction error (RPE) *R_t_* − *Q_t_*(*s_t_*, *a_t_*), which is the difference between the expected and actual reward at time *t* (Equation 1):
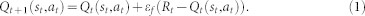
Our learning model contains four free parameters: a learning rate (*ε_f_*), reward sensitivity (*δ_f_*), value-independent bias for the choice of ¥100 (*a_t_*), (*b_f_*(*a_t_*)), and an exploration parameter (*β_f_*). The learning rate controls the effects of the RPE, and reward sensitivity transforms the actual reward (*r_t_*) in yen into a subjective reward (*R_t_*) for each participant (Equation 2):
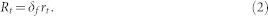
In relation to behavioural choice (Equation 3), the bias term represents a value-independent bias or inclination towards the choice of ¥100, and the exploration parameter controls how deterministically the value function leads to an advantageous behaviour:
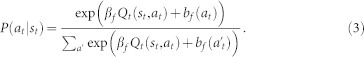
We estimated each participant's free parameters (denoted as the vector *θ*) from their trial-by-trial learning using the maximum likelihood-estimation method, which minimises the negative log-likelihood of the participant's behaviour (*D*), as shown in equations 4 and 5. This non-linear minimisation of equation 4 was conducted using the MATLAB function “fmincon”.
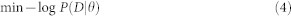

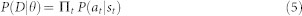
The probability of choosing an action, *a_t_* (¥100 or ¥1), given a visual cue, *s_t_*, was computed based on equation 3.

We evaluated the significance of each parameter using Akaike information criteria (AIC) and Bayesian information criteria (BIC) by comparing four models using the learning rate and exploration parameter (*εβ*), *εβ* with reward sensitivity (*εβδ*), *εβ* with ¥100 bias (*εβb*), and *εβ* with both reward sensitivity and ¥100 bias (*εβδb*). We calculated these information criteria for each participant and compared the mean scores (n = 91).

## Author Contributions

N.W. and M.H. designed the experiments. N.W. performed the experiments. N.W. and M.H. analysed the data, and wrote and reviewed the manuscript.

## Supplementary Material

Supplementary Information Supplementary figure

## Figures and Tables

**Table 1:** Descriptive statistics for participants in the four presentation conditions

	0.027 s	0.033 s.	0.040 s.	0.047 s.	*Statics*	*df*	*p*
Num. of participants	20	20	31	20	-	-	-
Sex ratio (Male/All)	0.80	0.65	0.74	0.60	*χ^2^* = 0.424	3	0.935
Mean Age (SD)	22.20	21.20	21.48	21.20	*F* = 1.420	3, 87	0.242
	(2.66)	(1.47)	(1.41)	(1.17)			
Mean clock time (hh:mm:ss) (SD)	12:51:00	12:42:00	12:32:54	13:18:00	*F* = 0.322	3, 87	0.810
	(2:50:00)	(2:50:52)	(2:34:33)	(2:27:06)			
Mean university/department academic score (SD)	54.63	56.50	56.21	54.38	*F* = 1.955	3, 87	0.127
	(4.95)	(2.78)	(2.90)	(3.34)			

Note: Mean clock time indicates the mean time at which a participant began the experiment. Mean university/department academic score was calculated as the mean academic ranking within the university department to which each participant belonged (the mean intelligence level across Japanese universities is standardised to 50).

## References

[b1] Kahneman D. & Tversky A. Prospect Theory - Analysis of Decision under Risk. Econometrica 47, 263–291 (1979).

[b2] McGaugh J. L. Memory consolidation and the amygdala: a systems perspective. Trends Neurosci 25, 456–461 (2002). 12183206 10.1016/s0166-2236(02)02211-7

[b3] Watanabe N., Sakagami M. & Haruno M. Reward prediction error signal enhanced by striatum-amygdala interaction explains the acceleration of probabilistic reward learning by emotion. J Neurosci 33, 4487–4493 (2013). 23467364 10.1523/JNEUROSCI.3400-12.2013 PMC6704947

[b4] Murphy S. T. & Zajonc R. B. Affect, cognition, and awareness: affective priming with optimal and suboptimal stimulus exposures. J Pers Soc Psychol 64, 723–739 (1993). 8505704 10.1037//0022-3514.64.5.723

[b5] Tsushima Y., Sasaki Y. & Watanabe T. Greater disruption due to failure of inhibitory control on an ambiguous distractor. Science 314, 1786–1788 (2006). 17170308 10.1126/science.1133197

[b6] Yotsumoto Y. *et al.* Performance Dip in motor response induced by task-irrelevant weaker coherent visual motion signals. Cereb Cortex 22, 1887–1893 (2012). 21940704 10.1093/cercor/bhr270 PMC3388893

[b7] Newell B. R. & Shanks D. R. Unconscious influences on decision making: a critical review. Behav Brain Sci 37, 1–19 (2014). 24461214 10.1017/S0140525X12003214

[b8] Karremans J. C., Stroebe W. & Claus J. Beyond Vicary's fantasies: The impact of subliminal priming and brand choice. J Exp Soc Psychol 42, 792–798 (2006).

[b9] Hassin R. R., Ferguson M. J., Shidlovski D. & Gross T. Subliminal exposure to national flags affects political thought and behavior. Proc Natl Acad Sci U S A 104, 19757–19761 (2007). 18056813 10.1073/pnas.0704679104 PMC2148371

[b10] Whalen P. J. *et al.* Masked presentations of emotional facial expressions modulate amygdala activity without explicit knowledge. J Neurosci 18, 411–418 (1998). 9412517 10.1523/JNEUROSCI.18-01-00411.1998 PMC6793390

[b11] Morris J. S., Ohman A. & Dolan R. J. A subcortical pathway to the right amygdala mediating “unseen” fear. Proc Natl Acad Sci U S A 96, 1680–1685 (1999). 9990084 10.1073/pnas.96.4.1680 PMC15559

[b12] Hannula D. E., Simons D. J. & Cohen N. J. Imaging implicit perception: promise and pitfalls. Nat Rev Neurosci 6, 247–255 (2005). 15738960 10.1038/nrn1630

[b13] Tamietto M. & de Gelder B. Neural bases of the non-conscious perception of emotional signals. Nat Rev Neurosci 11, 697–709 (2010). 20811475 10.1038/nrn2889

[b14] Liddell B. J. *et al.* A direct brainstem-amygdala-cortical ‘alarm' system for subliminal signals of fear. Neuroimage 24, 235–243 (2005). 15588615 10.1016/j.neuroimage.2004.08.016

[b15] Williams L. M. *et al.* Mode of functional connectivity in amygdala pathways dissociates level of awareness for signals of fear. J Neurosci 26, 9264–9271 (2006). 16957082 10.1523/JNEUROSCI.1016-06.2006 PMC6674508

[b16] Pessiglione M. *et al.* Subliminal instrumental conditioning demonstrated in the human brain. Neuron 59, 561–567 (2008). 18760693 10.1016/j.neuron.2008.07.005 PMC2572733

[b17] Haruno M., Kimura M. & Frith C. D. Activity in the nucleus accumbens and amygdala underlies individual differences in prosocial and individualistic economic choices. J Cog Neurosci 26, 1861–1870 (2014). 10.1162/jocn_a_00589 24564471

[b18] Epstein L. G. A definition of uncertainty aversion. Rev Econ Stud 66, 579–608 (1999).

[b19] Talmi D., Seymour B., Dayan P. & Dolan R. J. Human pavlovian-instrumental transfer. J Neurosci 28, 360–368 (2008). 18184778 10.1523/JNEUROSCI.4028-07.2008 PMC2636904

[b20] Bray S., Rangel A., Shimojo S., Balleine B. & O'Doherty J. P. The neural mechanisms underlying the influence of pavlovian cues on human decision making. J Neurosci 28, 5861–5866 (2008). 18509047 10.1523/JNEUROSCI.0897-08.2008 PMC6670800

[b21] Tottenham N. *et al.* The NimStim set of facial expressions: judgments from untrained research participants. Psychiatry Res 168, 242–249 (2009). 19564050 10.1016/j.psychres.2008.05.006 PMC3474329

[b22] Fleming S. M. & Frith C. D. The Cognitive Neuroscience of Metacognition (Springer, 2014).

[b23] Sutton R. S. & Bart A. G. Reinforcement learning (MIT Press, 1998).

[b24] Daw D. N. [Trial-by-Trial Data Analysis Using Computational Models.]. Decision Making, Affect, and Learning: Attention and Performance XXIII [Delgado, M. R., Phelps, E. A. & Robbins, T. W. (eds.)] [3–38] (Oxford Univ Press, 2011).

