# Supplementary material for: Effects of subconscious and conscious emotions on human cue–reward association learning
Source: Sci Rep. 2015 Feb 16;5:8478. doi: 10.1038/srep08478 (PMC4329552; doi:10.1038/srep08478)
Supplement: Supplementary Information — Supplementary figure [file srep08478-s1.pdf]

Effects of subconscious and conscious emotions on human cue–reward association learning

Noriya Watanabe[1, 2, 3], Masahiko Haruno[1, 4]*

Affiliations:

[1]Center for Information and Neural Networks, National Institute of Information and Communications Technology, Suita, Osaka 565-0871, Japan

[2]Japan Society for Promotion of Science

[3]Graduate School of Environmental Studies, Nagoya University

[4]Japan Science and Technology Agency

***Correspondence:**

Dr. Masahiko Haruno
NICT Cinet 1-4 Yamadaoka
Suita, Osaka 565-0871
Japan

Tel: +816-6879-4428
E-Mail: mharuno@nict.go.jp

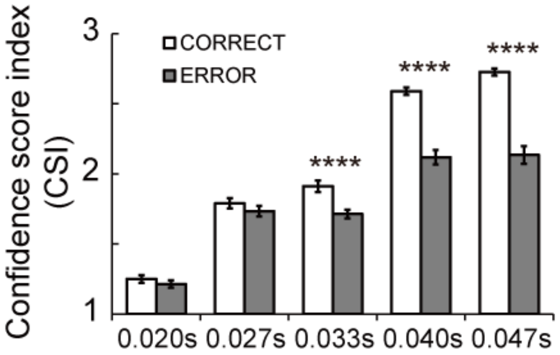

Supplementary Figure S1. Confidence score indices sorted by discrimination performance.

The results of confidence score index (CSI) were sorted based on discrimination performance. This analysis showed that the CSI in the 0.020 s and 0.027 s conditions were not statistically different between correct and error trials (paired t-test, 0.020 s: $t_{(90)} = 1.577$, $p \approx 1.000$, 0.027 s: $t_{(90)} = 1.549$, $p \approx 1.000$ with BC), but were significantly different in other conditions (paired t-test, 0.033 s: $t_{(90)} = 6.012$, $p < 0.0001$, 0.040 s: $t_{(90)} = 8.981$, $p < 0.0001$, 0.047 s: $t_{(90)} = 8.564$, $p < 0.0001$ with BC).
